# Socially adaptive cognitive architecture for human-robot collaboration in industrial settings

**DOI:** 10.3389/frobt.2024.1248646

**Published:** 2024-06-10

**Authors:** Ismael T. Freire, Oscar Guerrero-Rosado, Adrián F. Amil, Paul F. M. J. Verschure

**Affiliations:** Donders Institute for Brain, Cognition and Behaviour Radboud University, Nijmegen, Netherlands

**Keywords:** cognitive architecture, social robotics, human-robot collaboration, industry 4.0, distributed adaptive control

## Abstract

This paper introduces DAC-HRC, a novel cognitive architecture designed to optimize human-robot collaboration (HRC) in industrial settings, particularly within the context of Industry 4.0. The architecture is grounded in the Distributed Adaptive Control theory and the principles of joint intentionality and interdependence, which are key to effective HRC. Joint intentionality refers to the shared goals and mutual understanding between a human and a robot, while interdependence emphasizes the reliance on each other’s capabilities to complete tasks. DAC-HRC is applied to a hybrid recycling plant for the disassembly and recycling of Waste Electrical and Electronic Equipment (WEEE) devices. The architecture incorporates several cognitive modules operating at different timescales and abstraction levels, fostering adaptive collaboration that is personalized to each human user. The effectiveness of DAC-HRC is demonstrated through several pilot studies, showcasing functionalities such as turn-taking interaction, personalized error-handling mechanisms, adaptive safety measures, and gesture-based communication. These features enhance human-robot collaboration in the recycling plant by promoting real-time robot adaptation to human needs and preferences. The DAC-HRC architecture aims to contribute to the development of a new HRC paradigm by paving the way for more seamless and efficient collaboration in Industry 4.0 by relying on socially adept cognitive architectures.

## 1 Introduction

The increasing automation in the industry over the past decades has been partially driven by the adoption of robots, which have proven to be valuable tools for handling heavy, risky, and repetitive tasks. By automating these tasks, robots have helped alleviate the burden on human workers, contributing to improved safety, efficiency, and productivity Mazachek (2020) and Cette et al. (2021). However, robotic solutions have their own limitations; they tend to have restricted operational capacity in terms of degrees of freedom and decision-making and therefore they do not perform well outside of highly controlled and structured environments Vysocky and Novak (2016). As a result, complete automation might be neither feasible nor desirable Charalambous et al. (2015), Weiss et al. (2021), a discussion further amplified by recent advances in AI Verschure et al. (2020).

Instead, the future of Industry 4.0 Lasi et al. (2014) lies in the collaboration between humans and robots, capitalizing on the strengths of both in a manner that is both beneficial for the health and wellbeing of the workforce and productive for companies. Human-Robot Collaboration (HRC) is poised to become a key component of Industry 4.0 Baratta et al. (2023), with the primary goal of creating a safe environment for humans and robots to collaborate effectively. This transition from traditional automation to Industry 4.0 will be marked by a transformation involving the use of the latest advancements in information and communication technologies (ICTs) Robla-Gómez et al. (2017).

However, the transition to HRC in industrial settings faces several technical and scientific challenges Inkulu et al. (2021). Physically close collaboration between human workers and industrial robots has been limited so far, primarily due to safety concerns, such as potential collisions causing injury to human operators Robla-Gómez et al. (2017), Vysocky and Novak (2016). Recent advances in collaborative robotics, including the emergence of cobots, now allow for safer and closer interactions between humans and robots Weiss et al. (2021). Other technological advances come from transformative ICTs like artificial intelligence (AI) and the related field of machine learning (ML). In Semeraro et al. (2023), the authors review the impact of AI and ML in HRC, highlighting the shift towards cobots (collaborative robots) designed for safe, close-proximity work with humans. The authors emphasize the potential of ML to improve HRC by enabling robots to better understand and adapt to human behavior. Most approaches have relied on vision-based ML to handle objects and perform collaborative assembly in a safe manner. In addition, neural networks have been used to recognize human actions for the robot to assist the human when needed. Furthermore, reinforcement learning has shown great promise in decision-making during collaborative tasks that require outcome-dependent switching between the human and the robot. An example of an AI-based HRC solution in an industrial setting is outlined in Dimitropoulos et al. (2021), where the authors introduce an AI system with three modules to enable seamless human-robot collaboration by understanding the environment and operator actions, providing customized support, and adapting robot poses for better ergonomics, demonstrated through an elevator manufacturing case study. However, despite all these advances, Lemaignan et al. (2017) point out that advanced HRC will actually require robots to possess more advanced cognitive capabilities, such as common sense reasoning for context-aware decision making, which is not achievable yet. These advances in collaborative technologies call for novel paradigms to design collaboration in hybrid industrial settings that are in line with the ambitions of Industry 4.0.

One prime example of such a setting has been the implementation of a hybrid human-robot system in a recycling plant for Waste Electrical and Electronic Equipment (WEEE) products under the umbrella of the EU-funded HR-Recycler project Axenopoulos et al. (2019). This type of environment poses a unique set of challenges for human-robot interaction (HRI) and collaboration (HRC) Robla-Gómez et al. (2017), Vysocky and Novak (2016).

• Noise. Tasks carried out in a recycling plant involve actions such as hammering, cutting, grinding, and transporting heavy pallets, which makes the workplace a highly noisy environment where verbal communication is hampered if not completely disrupted.
• Task hazardousness. WEEE material disassembly involves the manipulation of sharp and heavy materials that during their processing may produce hazardous metal shavings and sparks during processing. To ensure the integrity of the workers, WEEE recycling requires safety measures such as wearing Personal Protective Equipment and maintaining a large distance between co-workers that, at the same time, limits the collaboration between counterparts.
• Dynamic environment. Plant configuration constantly evolves. The continuous processing of WEEE material involves piles of WEEE devices disappearing and new piles arriving at different locations, workers leaving their workbenches to attend to other assignments, surfaces getting covered by metallic dust, light conditions changing along the day, etc. This prevents cobots from following fixed routines.

In parallel, the specific tasks involved in the recycling process demand physically close collaboration and interaction between workers and robots, requiring human-robot collaboration to be socially adaptive.

To achieve social adaptability, HR-Recycler builds on the new dimension that human-robot collaboration (HRC) takes in Industry 4.0, becoming a complex sociotechnical system where agency—the capacity to act—is not solely attributed to humans. Instead, it is shared among humans and non-human agents, such as machines, robots, sensors, and software Weiss et al. (2021). This paradigm shift is crucial as it acknowledges the increasing demand for more interactive roles between humans and cobots within industrial settings and, therefore, the need to develop new control systems that accommodate this emergent reality. Additionally, this shift also highlights the rise of novel configurations of shared control and distributed agency, which are key aspects of this new industrial paradigm.

To address the challenges posed by Industry 4.0, including the integration of collaborative robots (cobots) in hybrid industrial environments, this paper introduces a novel systems-level control paradigm for designing and implementing cognitive architectures tailored for Human-Robot Collaboration (HRC). Accordingly, we present DAC-HRC, a novel cognitive architecture that is specifically designed to facilitate socially adaptive human-robot collaboration within industrial contexts. In the next sections of the introduction, first, we outline the key principles for human-robot collaboration upon which the cognitive architecture is based, with and special emphasis on the notions of joint intentionality and interdependence. We then introduce the Distributed Adaptive Control perspective for building HRC and highlight how each of the specialized modules of the DAC-HRC cognitive architecture is related to these principles and the state-of-the-art of each implemented functionality.

### 1.1 Principles for human-robot collaboration in industrial settings

To develop an effective socially adaptive cognitive architecture within the context of a hybrid recycling plant, we reviewed state-of-the-art Human-Robot Collaboration principles for industrial settings aiming to conciliate the perspective of different authors that have explored this in the past. As a result, the following principles were considered:

• Implicit Switch Modes: The system must fluidly alternate between various interaction modes, adapting to the human worker’s context without burdening them Bauer et al. (2008).
• Natural Cues: Intuitive interaction is facilitated by leveraging humans’ inherent understanding of natural signals, enabling humans to communicate with robots using familiar gestures and symbols Goodrich and Olsen (2003).
• Direct World Manipulation: Interactions are designed to serve the ultimate purpose of task completion in a tangible world, allowing humans to directly influence robotic behavior to navigate the unpredictable physical environment of industrial settings Adams (2005).
• Information Manipulation: Information presented by the robot must be actionable, supporting the human worker’s decision-making processes and promoting goal-oriented collaboration Goodrich and Olsen (2003).
• Attention Management: The design of HRC interactions should cater to the cognitive limitations of human attention, ensuring that critical information is highlighted and that potential attentional lapses are mitigated Adams (2005).
• Situational Awareness: Maintaining an acute awareness of the robot’s internal and external state is paramount, enabling human workers to anticipate robotic actions and intervene when necessary Goodrich and Olsen (2003).
• Safety: Paramount to any HRC system is the unwavering commitment to human safety, ensuring that robots can navigate the potential hazards of industrial tasks without endangering human collaborators Goodrich and Olsen (2003).

#### 1.1.1 Joint intentionality and interdependence as core principles for industrial HRC

Beyond the general HRI principles described above, the DAC-HRC architecture incorporates two core principles coming from our current understanding of the origins of human collaboration: interdependence and joint intentionality.

Joint intentionality refers to the shared mental states and cooperative activities that arise when individuals engage in collaborative endeavors. Research on social cognition posits that shared intentionality is a unique feature of human cognition, setting us apart from other primates Tomasello et al. (2005). It manifests in the form of shared goals, joint attention, and mutual knowledge among individuals working together. For instance, when two people collaborate to lift a heavy object, they share a common goal (i.e., moving the object) and are aware of each other’s intentions, roles, and actions.

This concept is particularly relevant in the context of Human-Robot Collaboration (HRC), as it emphasizes the importance of mutual understanding, communication, and coordination between human workers and their robotic partners. In industrial HRC, developing systems capable of exhibiting joint intentionality is essential for ensuring more efficient and safer interactions between human workers and robots. In this context, developing shared intentionality in artificial and hybrid collaborative systems would imply the ability to (1) detect and predict human intentions, actions, and goals, (2) communicate its intentions, actions, and goals to human workers and (3) coordinate and adapt its behavior based on the shared goals and the feedback from the ongoing collaboration.

Interdependence is another foundational aspect of current theories of the evolution of human cooperation Tomasello et al. (2012). It refers to the reliance of individuals on one another to achieve shared goals or complete tasks, and its key role in vital tasks for early humans such as obligate collaborative foraging Tomasello (2009), O’Madagain and Tomasello (2022). Applied to the context of HRC, interdependence implies that both human workers and robots depend on each other’s actions, skills, and knowledge to execute tasks effectively. The Interdependence Hypothesis Tomasello et al. (2012) suggests that interdependence fosters cooperation, as it encourages individuals to align their goals, share information, and coordinate their actions.

In HRC, task interdependence between humans and robots can motivate the design of systems that (1) recognize the skills and capabilities of human workers and adapt their behavior accordingly, (2) share task-related information with human workers, facilitating mutual understanding and efficient task execution (3) respond to changes in the task or environment, adjusting their actions to maintain effective collaboration.

To illustrate, consider a collaborative robot in an automotive assembly line. The cobot could be designed to recognize the specific skills of the human worker, such as their proficiency in installing certain parts. Based on this recognition, the cobot could adapt its behavior to complement the worker’s skills, perhaps by preparing the necessary parts or tools for the worker’s next task. Moreover, the cobot could share task-related information with the worker, such as the sequence of assembly steps or the status of the parts supply, facilitating mutual understanding and efficient task execution. Finally, the cobot should be able to respond to changes in the task or environment. For instance, if a part is missing or defective, the cobot could adjust its actions, perhaps by fetching a replacement part or alerting the worker to the issue.

In understanding and implementing these core principles of joint intentionality and interdependence, it becomes apparent that a sophisticated cognitive architecture is required: one that not only comprehends human social behaviors but also adapts and responds to the dynamic nuances of industrial settings. This necessity brings us to the Distributed Adaptive Control (DAC) approach, which provides a validated and biologically-inspired framework. The DAC approach, with its layered control system and emphasis on adaptability, is ideally suited to embed these principles into the fabric of human-robot collaboration. As we transition to exploring the DAC-HRC architecture, we will see how each of its specialized modules is designed to operationalize the principles of joint intentionality and interdependence, thereby creating a harmonious and effective collaborative environment between humans and robots in industrial settings.

### 1.2 The distributed adaptive control approach to human-robot collaboration

A cognitive architecture is a modular control system that governs a robot’s decision-making, information processing, and environmental interaction Vernon (2022), Moulin-Frier et al. (2017). It is from the interaction and interdependence of its constituent modules that a cognitive architecture displays cognitive capabilities such as perception, decision-making, memory, or social learning. The Distributed Adaptive Control (DAC) theory of mind and brain Verschure, (2012) offers a robust theoretical foundation for such architectures, as it has been previously shown in various HRI scenarios Lallée et al. (2015), Moulin-Frier et al. (2017), Fischer et al. (2018). DAC views the brain as a hierarchical system with multiple control layers, each crucial for adaptive behavior in diverse physical and social contexts Verschure, (2012), Verschure et al. (2014). This biologically grounded modular modeling approach is especially suitable for addressing the HR-Recycler environment’s challenges, which demand adaptive and goal-oriented actions.

Informed by the DAC framework, the DAC-HRC architecture we introduce in this paper integrates four specialized modules that reflect key principles for effective HRC. Each module is tailored to specific principles, forming a cohesive and operational control system:

• Task Planner: Coordinates the proper disassembly steps for each device, organizes the disassembly procedure and the turn-taking between human and robot actions, centralizes task-related information among the DAC-HRC modules, and implements safe and robust error-handling protocols. The Task Planner reflects the principles of shared intentionality and interdependence, as it involves a mutual understanding between the human and robot about the sequence of tasks and reliance on each other’s capabilities to complete these tasks. Moreover, by orchestrating robot control and human-robot interaction, it also embodies the principles of ’implicit switch modes’ and ’direct world manipulation’.
• Interaction Manager: Serves as a multimodal, non-verbal communication interface, facilitating efficient communication and interaction between humans and robots. To achieve this, the module integrates multimodal channels of communication, ranging from audiovisual interfaces such as tablets to embodied gesture-based, communication. By handling natural embodied human-robot interaction based on gestures and adapting to the context of the information visualized on tablet devices, this module implements the principles of ’natural cues’, ’attention management’, ’information manipulation’, and ’situational awareness’. By jointly visualizing in the tablet device the progress and information about the status of the human worker and the robot, this module also creates a sense of shared intentionality.
• Socially Adaptive Safety Engine: Acts as a context-aware adaptive safety mechanism, controlling the safety distances between humans and robots as well as the speed of the interactions, adapting them to the context and the preference of the human co-worker. It deals with the integration of the relevant environmental, social, and material information that comes from other modules to adapt the safety mechanisms of the human-robot collaboration, directly addressing the principles of ’safety’ and ’situational awareness’. It dynamically adjusts robot behavior to align the safety measures with human preferences and the task context, also emphasizing the principle of interdependence.
• Worker Model: Creates an internal model of human workers, focusing on the principles of ’information manipulation’, ’implicit switch modes’, ’situational awareness’, and ’interdependence’. This module handles information about the human worker, using it to adapt the robot’s behavior in alignment with the worker’s preferences. This module is instrumental in adaption the overall collaboration schemes to the human worker, enabling the robot to adjust its actions and fostering a collaborative relationship where both parties rely on and benefit from each other’s strengths.

By integrating these modules within a single cognitive architecture, DAC-HRC, we create a robust control system for HRC in industrial settings. This system is inherently socially adaptive, as it is capable of dynamically adjusting in real-time to accommodate the varied preferences of human workers and the nuances of different scenarios. Moreover, it facilitates mutual understanding and fosters effective collaboration between human workers and robots, a critical requirement for addressing the complex tasks encountered in the HR-Recycler’s recycling plant use case. Comprising various specialized modules, the DAC-HRC cognitive architecture implements distinct functions, each grounded in contemporary, state-of-the-art solutions derived from the literature.

#### 1.2.1 Task planner as a hierarchical finite state machine

Task planners play a pivotal role in robotics, especially in enabling robots to adeptly navigate complex and unpredictable environments. In domains like electronic waste recycling operations, the ability of robots to perform a range of tasks, from sorting to processing diverse types of devices and components, hinges on sophisticated task planning mechanisms Alami et al. (2005). The cornerstone of contemporary task planning in robotics is the use of finite-state machines (FSMs), revered for their simplicity and intuitiveness in modeling robot behavior amidst uncertainty Foukarakis et al. (2014).

Finite-state machines are essentially mathematical constructs encompassing a finite set of states, transitions between these states, and corresponding input/output events. This structure empowers robots with the ability to efficiently adapt their behavior in response to varying conditions, a feature crucial in the fluctuating environment of a recycling plant. The inherent simplicity of FSMs, however, can be a limitation when dealing with more complex behaviors.

To address this complexity, hierarchical finite-state machines (HFSMs) have emerged as a potent solution to orchestrate complex robot behaviors. HFSMs represent behaviors in a layered structure of FSMs, where each level corresponds to a specific subtask or behavior component Johannsmeier and Haddadin (2016). This hierarchical arrangement facilitates a modular and scalable approach to task planTask Planner, the Socially Adaptive Safety Engine, the Worker Model and the Interaction Managerning. By breaking down overall robot behavior into manageable subtasks, HFSMs offer a tailored solution to the multifaceted tasks encountered in electronic waste recycling. This approach not only enhances the robot’s efficiency and adaptability but also allows for easier integration and updates to the task planning system as recycling requirements evolve.

Moreover, the incorporation of human-in-the-loop methodologies in task planning signifies a significant evolution in robotic systems. This approach involves integrating human feedback and inputs directly into the robot’s control mechanism, enabling a more dynamic and adaptable interaction between humans and robots Raessa et al. (2020). In the context of electronic waste recycling, this means that robots can be more responsive to human operator’s preferences and needs, thereby enhancing collaboration efficiency and safety.

In implementing HFSMs, the Task Planner module within the DAC-HRC cognitive architecture embodies these principles, leveraging the hierarchical structure to manage complex tasks while remaining adaptable to the diverse challenges presented in electronic waste recycling. The module’s design allows for seamless incorporation of human inputs, ensuring that the robotic system is not only responsive but also attuned to the needs and preferences of different human workers. This integration of advanced HFSMs within the DAC-HRC architecture illustrates a commitment to developing robotic systems that are both technically proficient and collaboratively effective in complex industrial settings.

#### 1.2.2 Interaction manager as a multimodal non-verbal communication protocol

The DAC-HRC architecture’s Interaction Manager advances the paradigm of multi-modal non-verbal communication, pivotal for intuitive and effective human-robot collaboration in industrial settings. In the human-centered HRI paradigm, an essential aspect of implementing a successful and effective HRI is building a natural and intuitive interaction Wang et al. (2022). In recognition of the importance of non-verbal communication modalities, particularly in noisy industrial settings, the Interaction Manager eschews auditory channels in favor of gesture-based and tablet-based interfaces.

Gestures serve as a fundamental form of human communication, making them ideal for conveying rapid commands in human-robot interaction (HRI) Vouloutsi et al. (2020), Pezzulo et al. (2019). Gesture-based communication harnesses the natural propensity for humans to use physical gestures, thereby facilitating a more immediate and universal form of interaction Liu and Wang (2018), Wang et al. (2022), Peral et al. (2022). The Interaction Manager incorporates a repertoire of shape-constrained gestures Alonso-Mora et al. (2015) tailored to the communication needs specific to the HR-Recycler’s project, which facilitates natural and intuitive interactions without extensive training Vouloutsi et al. (2020) while also ensuring accurate recognition and interpretation by the robotic agents Peral et al. (2022).

To complement gesture-based interactions and cater to scenarios necessitating more detailed information exchange, the architecture also integrates tablet-based communication. This method leverages interactive applications, which, while commonly used for teleoperation Yepes et al. (2013), Best and Moghadam (2014), Luz et al. (2019), are repurposed in the DAC-HRC to enhance the human-robot bond and situational awareness Goodrich and Olsen (2003). The tablet application provides a direct interface for the human worker to receive updates on the robot’s internal state and environmental interpretations, aligning with key HRI principles Goodrich and Olsen (2003), Adams (2005).

The combined use of gesture and tablet-based interactions by the Interaction Manager represents a state-of-the-art approach to non-verbal HRI. It successfully navigates the challenges of noisy industrial settings, where traditional verbal communication is untenable, and establishes a robust, adaptive, and user-friendly communication system conducive to the dynamic requirements of the HR-Recycler’s operations.

#### 1.2.3 Socially adaptive safety engine as an allostatic control system

A robot must not endanger a human under any circumstances. This premise, already formulated by Isaac Asimov in his famous “Three Laws of Robotics,” is crucial for any robotic installation but especially for those promoting interaction and collaboration between humans and synthetic agents Asimov, (2004). Importantly, in industrial settings interactions must be designed bearing in mind that robots occasionally will represent a source of danger to humans because of the tools they employ to perform hazardous tasks such as cutting, hammering, or moving heavy objects. Safety measures need to be implemented both according to the work to be performed and the human demands Van Wynsberghe (2020).

In the context of the WEEE recycling factory, humans and robots have to interact with a variety of different objects and tools and realize many changing sequences of actions in order to successfully complete their tasks Axenopoulos et al. (2019). Although each of the robotic components has its own built-in safety mechanisms and corresponding certified ISO safety measures, the interaction of all these elements together will require an additional layer of control that can adapt their behavior to the requirements of the hybrid recycling plant while following human-centered design principles. This layer of control is the Socially Adaptive Safety Engine (SASE).

The Socially Adaptive Safety Engine within the DAC-HRC architecture goes beyond mere harm avoidance to actively promote cooperation Freire et al. (2020a). The SASE not only adheres to basic safety principles but also engages in more flexible adaptation of the robot’s behavior to the preferences of the human worker, thus fostering a more cooperative and harmonious human-robot interaction. It also reflects the principles of shared intentionality and interdependence, as it involves a mutual understanding between the human and robot about the safety measures and reliance on each other’s capabilities to maintain safety during the disassembly process.

The goal of the SASE is to promote human-robot cooperation by building safer, more trustworthy, and personalized interactions with human users Kok and Soh (2020), Christoforakos et al. (2021). It does so by regulating and adapting the robot’s behavior to the particular human preferences of every user Senft et al. (2019), Edmonds et al. (2019). In this way, it also serves as an extra layer of security for the system by integrating contextual information from the environment and using it to prevent potentially harmful situations Yang et al. (2018). At the heart of this approach is the implementation of an allostatic control system Sanchez-Fibla et al. (2010), Guerrero Rosado et al., 2022. This system aims to ensure harm avoidance and promote cooperative behaviors, which are two fundamental aspects of ethical machine behavior Freire et al. (2020a).

In essence, the Socially Adaptive Safety Engine encapsulates the ethos of the DAC-HRC architecture—prioritizing human safety and introducing dynamic adaptability, thereby exemplifying a model of responsible and responsive artificial intelligence in industrial settings.

#### 1.2.4 Worker model based on human-centered design principles

User modeling systems rely on data gathering to create user models, either explicitly or implicitly Luna-Reyes and Andersen (2003). The integration of novel machine learning techniques has significantly enhanced the capabilities of these systems, steering them towards more data-driven strategies Kontogianni et al. (2018). One emerging technique being implemented in these data-driven user modeling practices is the Digital Twin concept, which generates or collects digital data representing a physical entity, emphasizing the connection between the physical and virtual counterpart through real-time information flow Bruynseels et al. (2018), Negri et al. (2017).

Digital Twin technologies have been applied in various contexts, such as healthcare Croatti et al. (2020) and human-robot interaction Wilhelm et al. (2021), Malik and Brem (2021), Wang et al. (2020). In industrial settings, Digital Twins have been utilized for tasks like interactive welding, bridging human users and robots through bidirectional information flow, and benefiting novice welder training Wang et al. (2020), Jokinen and Wilcock (2015). However, these data-driven approaches raise concerns regarding big data management, privacy, and trustworthiness, especially when applied to sensitive fields Kumar et al. (2020). The Human-Centered AI paradigm aims to address these concerns by prioritizing methodologies that meet user needs while operating transparently, delivering equitable outcomes, and respecting privacy Xu (2019), Riedl (2019). This approach also aligns with legislation such as the European General Data Protection Regulation (GDPR) Kloza et al. (2019).

The Worker Model module of the DAC-HRC cognitive architecture follows such human-centered design principles by maximizing functionality while minimizing the amount of data gathered from the user Xu (2019), Riedl (2019). This design strategy ensures that the Worker Model respects user privacy while still providing effective support for human-robot collaboration in the disassembly of WEEE devices. The main goal of the Worker Model is to collect, process, and store all the relevant information regarding each user of the system and integrate it into one single, coherent data structure. This information is used by the DAC-HRC architecture to flexibly adapt the human-robot collaboration paradigm to the human partner. In other words, the Worker Model creates a virtualization of the human worker that allows the collaborative architecture to dynamically adjust its parameters to ensure a personalized interaction.

In essence, the Worker Model’s integration into the DAC-HRC architecture not only enhances the adaptability of the human-robot collaboration paradigm but also embodies a human-centric focus into the design of these new technologies Xu (2019), Riedl (2019).

The rest of the chapter is organized as follows: In the following section section, we first describe the aCell, a specific experimental setup designed for the collaborative disassembly of WEEE devices. We then continue describing in detail each of the components of the DAC-HRC architecture along with its interactions. We proceed with a report of the main results showcasing the functionalities of the architecture across the different tested use cases, and conclude with a discussion of the main outcomes of the study, its limitations, implications, and future work.

## 2 Methods

### 2.1 The aCell experimental setup

The experimental setup consists of a specific spatial and technical configuration of an adaptive Collaborative Cell (aCell) designed for the collaborative disassembly of Waste Electrical and Electronic Equipment (WEEE) devices. The concept of an aCell represents an evolution in the way we approach task allocation in HRC Axenopoulos et al. (2019). Traditional industrial HRI methodologies often focus on individual tasks within a single work cell, with the human and robot working in isolation on specific tasks. However, the aCell concept promotes a more holistic view of HRC that takes into account the interdependence between humans and robots. It envisions a dynamic, integrated system where the human and robot work together across multiple tasks, adapting to changes in the work environment and each other’s capabilities. This approach aims to enhance the overall efficiency and effectiveness of the collaboration, rather than optimizing individual tasks in isolation.

An aCell is a dynamic and adaptive component of a hybrid factory, responsible for a specific task and for a given time period. The responsibilities, resource allocation, and overall positioning of its elements within the factory are dynamically assigned and adapted in real time with respect to the overall factory workflow demands, available skills, and available resources. In the context of our study, the cell consists of a human worker collaborating with a cobot, with each of them possessing specific, known skills. They operate as part of a joint intentional team with shared goals: to disassemble a series of Waste Electrical and Electronic Equipment (WEEE) devices.

The design of the aCell is grounded in the interdependence and joint intentionality between the human worker and the cobot. The components of the aCell are interdependent since effective task completion requires the combination of both human and robot capacities while sharing the same goals for disassembly. By taking into account the complementary skills and shared goals of the human-cobot dyad, the aCell can be seen as a single collaborative unit whose control is distributed. The DAC-HRC architecture we present in this chapter is designed as a control system to deal with such hybrid collaborative entities, by orchestrating the disassembly process while also taking into account human workers’ safety, and promoting context and real-time adaptation in the dynamic and complex environment of the WEEE recycling plant.

**FIGURE 1:**
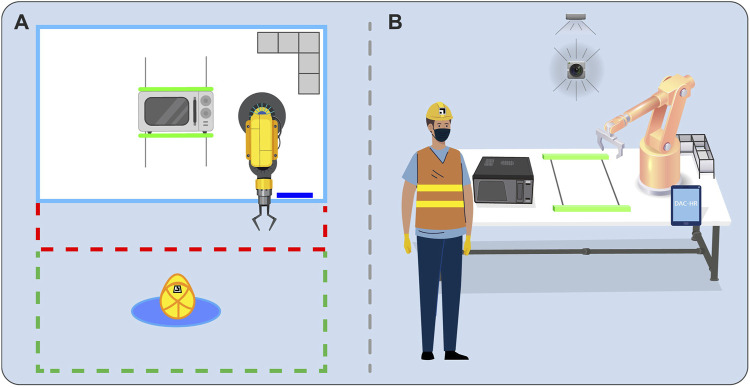
Experimental Set-up. **(A)** Cenital view of the aCell. Green dashed lines illustrate the human working area, being limited by a safety threshold (red dashed lines). **(B)** Complete configuration of the aCell including the human worker, the cobot, the WEEE device to be disassembled, the clamping tool, the tablet device where the Interaction Manager app is displayed, the cobot tool rack, and the cameras monitoring the behavior of both the human worker and robot.

In this work, the aCell is composed of two primary regions (Figure 1): the open space, where the human worker performs tasks without hindrance, and the workbench, where the DAC-HRC synthetic actuators are strategically located.

WEEE materials are positioned on the workbench for collaborative disassembly by the human worker and a COMAU Racer-5 collaborative robot (cobot). To further augment the functionality of the cobot, a tool rack is in place to house and arrange Racer-5 tools that are not currently in use. These tools include a screwdriver, a vacuum gripper, a finger gripper, and a cutting device.

Two vision modules allowed DAC-HRC to be informed by the aCell regarding the status of the disassembly task and the human worker. These vision modules were designed following state-of-the-art computer vision techniques Tran et al. (2018), Ghadiyaram et al. (2019), Cao et al. (2017), Kalitsios et al. (2022), Gabler and Wollherr (2022) and provided by other HR-Recycler partners. A vision system oriented towards the open space captures and processes information related to the human worker, such as their identity, position, and behaviors like gestures. To enable the cobot to gather information on the WEEE device, such as the status of its components, an additional vision system is directed toward the disassembly area, informing about the device’s state. A mechanical clamping tool is also integrated with the workbench to stabilize the WEEE device while either the human worker or cobot performs actions on it.

Lastly, the workbench, and by extension, the aCell, are supplemented by a tablet display that enables a bilateral communication channel between DAC-HRC and the human worker, displaying relevant information (e.g., current task status), and serving as a medium for human feedback.

### 2.2 The DAC-HRC cognitive architecture

The aim of the DAC-HRC architecture is to develop a robust human-robot collaboration control system for industrial settings that adapts to different workers through strategies learned from data obtained during the interaction. This process reflects the principles of shared intentionality, as it involves a mutual understanding between the human and the robot about the worker’s skills and preferences. It also illustrates the principle of interdependence, as the architecture relies on both the human and robot’s capabilities to ensure safe and efficient human-robot collaboration.

More concretely, DAC-HRC enables robotic components to tailor their interactions to the needs of their collaborative human partner, taking into account their unique skills, capabilities, and preferences. In order to achieve such a level of personalized adaptation to each human partner, each of its core four functionalities, control, safety, adaptation, and interaction are all distributed across the whole architecture, while having their specialized cognitive modules: the Task Planner, the Socially Adaptive Safety Engine, the Worker Model and the Interaction Manager, respectively.

DAC-HRC follows the design principles of the Distributed Adaptive Control theory, which states that the goal of cognition in embodied agents is to control action, and as such, any cognitive system can be described as a modular, hierarchical control system operating at different spatiotemporal timescales Verschure et al. (2012). The DAC theory can be expressed as a robot-based cognitive architecture organized in two complementary structures: layers and columns. The columnar organization defines the processing of states of the world, the self, and the generation of action. The organizational layers define the different levels of control, starting from the Soma Layer integrating all sensors and effectors of the system, the real-time reactive sensorimotor control in the Reactive Layer, the adaptive associative learning and allostatic control in the Adaptive Layer, up to abstract and symbolic manipulation and context-based control in the Contextual Layer.

**FIGURE 2:**
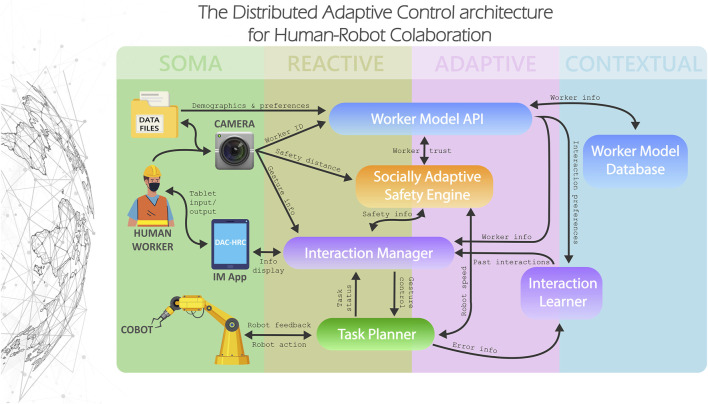
The DAC-HRC architecture for human-robot collaboration in industrial settings. DAC-HRC is structured in four layers of control (from left to right): Soma, Reactive, Adaptive, and Contextual; and composed of four specialized cognitive modules: the Task Planner, the Interaction Manager, the Socially Adaptive Safety Engine, and the Worker Model. The cognitive modules are distributed across various levels of the layered architecture, aligning with their control timescales and levels of information and sensory abstraction. The Soma Layer represents the physical components of the aCell, including the cameras, tablet, cobot, and human worker. The Reactive Layer houses the Interaction Manager and Task Planner modules, responsible for orchestrating human-machine communication and managing task allocation, respectively. The Socially Adaptive Safety Engine and the API component of the Worker Model, which adapts safety measures and processes real-time worker information, span both the Reactive and Adaptive Layers. The Contextual Layer is hThe effectiveness of DAC-HRCome to the Worker Model Database, storing long-term memory of past interactions and user preferences, and the Interaction Learner, which uses this information to adapt tablet display options based on past interactions.

DAC-HRC is organized following DAC’s layered structure, where each of its specialized HRC cognitive modules is located at different levels of the layered architecture based on their spatiotemporal timescales of control and their informational and sensory abstraction, as we can see in Figure 2. In other words, the cognitive modules are strategically distributed throughout the architecture based on the specific temporal and spatial requirements for control, as well as the degree to which they process and abstract sensory information and relevant data.

Its Soma Layer is defined by the hybrid combination of synthetic and biological sensors and actuators that comprise the aCell, that is, the cameras, the tablet, the cobot, and the human worker. In its reactive layer, DAC-HRC incorporates the Interaction Manager and the Task Planner modules. The Interaction Manager is devoted to the human-machine interaction protocols necessary to orchestrate communication between the human and the cobot. The Task Planner is in charge of the adequate task allocation among the members of the collaborative entity. It sequentially organizes the disassembly tasks and controls the correct turn-taking behavior between the human and the cobot. The Socially Adaptive Safety Engine, which is in charge of providing an additional layer of safety that adapts the security distances and robot speed to the particular preferences of the human partner and the current task context, spans both the reactive and adaptive layers. The same applies to the API component of the Worker Model, which deals with the real-time information related to the worker, as well as with the update of the Database. In the contextual layer, the Worker Model Database provides the system with an internal model of the human workers, storing in its long-term memory the past interactions between each user and the system, as well as relevant information for adapting the overall collaboration to the preferences of the human partner. The Interaction Learner, spanning both the contextual and adaptive layers, uses the contextual information to learn from past interactions with the system to adapt the options displayed by the Interaction Manager through the tablet device. In the following sections, we describe in detail the technical implementation of the cognitive modules of the DAC-HRC architecture.

#### 2.2.1 Task planner

**FIGURE 3:**
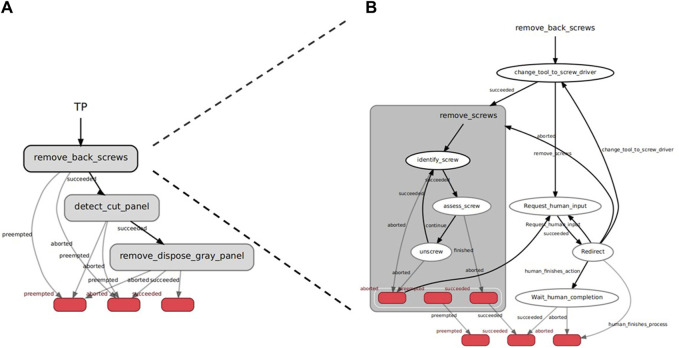
Task Planner. Visualization of the Device **(A)** and Task **(B)** levels of the Task Planner’s hierarchical finite-state-machine (HFSM) for the flat-panel display use case. **(A)** Device level of the Task Planner’s HFSM. This level showcases the finite-state machines responsible for sequentially connecting the disassembly steps (i.e., Tasks) required for the correct disassembly of the flat-panel display. **(B)** Task level of the Task Planner’s HFSM. At this level, dedicated error-handling mechanisms within the Task Planner engage the worker when errors occur during the execution of an action (in this case, ‘remove screws’). This ensures that the worker is actively involved in resolving any issues that may arise. Feedback and responses from the worker serve to redirect the state of the Task Planner, enabling effective error resolution and maintaining the overall flow of the task execution.

The DAC-HRC’s Task Planner module is conceived as a human-in-the-loop hierarchical finite state machine that encompasses all disassembly steps of all devices, as well as the error-handling protocols. The Task Planner (TP) has been developed to ensure robust orchestration of various components contributing to the disassembly of WEEE devices within the aCell system. The objectives of the TP are to coordinate the proper disassembly steps for each device, organize the disassembly procedure and robot-worker interleaving, centralize task-related information among the DAC-HRC modules, and implement safe and robust error-handling protocols. The TP reflects the principles of shared intentionality and interdependence, as it involves a mutual understanding between the human and robot about the sequence of tasks and reliance on each other’s capabilities to complete these tasks. The TP integrates and coordinates low-level sensorimotor information (coming from the computer vision and robotic components of the aCell) with high-level information about the task and the interaction (coming from the upper control layers of the architecture). Therefore, within the TP’s HFSM, we find states with different levels of abstraction and description. The Task Planner operates at five levels:

• Task Planner. This level corresponds to the highest level of abstraction, which contains the state machines (SM) of all 4 Devices. It also contains the functionalities that deal with continuous status reports, as well as direct human interactions (through the Interaction Manager, or IM) that allow the TP to be suddenly interrupted by the worker.
• Device. This level contains the state machine (SM) that links the steps (i.e., Tasks) needed for the proper disassembly of a particular device, in a sequential manner (i.e., without internal loops). Thus, it comprises a straightforward sequential SM with all the necessary steps or Tasks to be executed in the right order, steps which have been pre-defined based on domain-specific knowledge of the proper disassembly of the devices (see Figure 3A).
• Task. In this level, a particular Task–involving one or more Actions (see below)— is executed, with the end result of removing a particular component of the device (e.g., “top lid removal of the e-light”). Here, errors during the execution of an Action are handled in a dedicated SM so that the worker is engaged whenever needed (see Figure 3B). Feedback and responses from the worker redirect the state of the TP accordingly (e.g., if an error with the robot occurs and the worker decides to complete that Task themselves).
• Action. This is the atomic level of description, where specific modules are uniquely engaged via ROS communication (e.g., ROS-actions or ROS services). During an Action, either a ROS action is sent to a robot to perform a specific action (e.g., “change tool to vacuum gripper,” or “dispose lid”), or a ROS service is issued to the vision module to acquire the necessary information that the robot will need to perform a subsequent action (e.g., “identify the grasping pose of the lid”).
• Sub-action. In some cases where Actions need to be repeated several times and imply feedback loops with vision and the robot, an additional level is introduced so that the SM design becomes more modular and robust (e.g., “Unscrew the six screws of the microwave’s cover” is designed so that a dedicated SM to unscrew coordinating the robot and vision feedback can be called in loop until all screws have been removed).

The Task Planner is implemented with the Smach-ROS python library, which allows seamless integration of HFSMs with ROS-based communication protocols Bohren and Cousins (2010), Pradalier, (2017). Crucially, internal data structures allow the conveying of information received from vision (response of a ROS service) to the robot (goal of a ROS action). In an SM, the transitions between states depend on the outcome of each State after having been executed. An example of a Task can be seen in Figure 3. In general, an outcome “succeeded” will make the SM transition to the next Task or Action (depending on the level). The outcome “aborted” will engage the error-handling loop (see section Error Handling below), which asks the worker for feedback, and transitions to different states according to the worker’s decision (e.g., the robot tries again, or the worker finishes the Task and then the TP moves to the next Task). The hierarchical structure of the FMS can be achieved because all SMs are treated as States too, inheriting their properties.

#### 2.2.2 Interaction manager and interaction learner

The Interaction Manager module plays a vital role in facilitating efficient communication and interaction between humans and robots. To achieve this, the module integrates multimodal channels of communication, ranging from audiovisual interfaces to embodied non-verbal communication. To account for high levels of noise and equipment worn by workers, verbal communication was excluded from the communication repertoire. The two main modes of interaction, gesture-based communication, and tablet-based communication, have been chosen to address the noisy industrial environment and safety concerns during collaboration between human workers and robots.

Gesture-based communication provides a fast and direct means for the human worker to convey simple and fast control commands and responses to the robotic companion, making it a useful and naturalistic way of communicating in the collaborative environment of the aCell. The Interaction Manager integrates eight different communication signals, providing a rich set of gestures for effective communication between the worker and the robotic system.

aCell-to-human communication is enabled through a tablet-based application, representing the main communication channel through which the system can provide detailed information about the current task’s state. Additionally, through this Interaction Manager application (IM app), the system can request human intervention during the disassembly or request human input for problem-solving or decision-making when unforeseen problematic situations are faced.

These two main modes of interaction have been chosen to cover the speed-accuracy trade-off, with gestures for simpler but time-sensitive interactions, and tablets for slower but more fine-grained information exchange. This dual communication paradigm accommodates individual human preferences and ensures efficient collaboration in various human-robot collaboration scenarios, as we will see in the Results section.

The Interaction Learner adds a level of personalization on top of the Interaction Manager functionalities by providing it with an adaptive mechanism to support human-robot decision-making based on the prior history of interactions between the human and the cobot. Its main function is to keep track of human-robot interactions and human feedback during error-handling scenarios. It computes useful statistics based on the history of human-robot interactions, and when a similar situation is encountered, it adapts the options displayed in the tablet by the Interaction Manager in a way that enhances collaborative decision-making by highlighting on the menu the most frequently selected options by that worker in a given situation. This level of adaptation takes into account the human-robot interactions at each specific step during the disassembly and for each worker in particular.

#### 2.2.3 Socially adaptive safety engine

The design of the Socially Adaptive Safety Engine (SASE) incorporates a set of reactive control systems inspired by Pavlovian appetitive and aversive drives Freire et al. (2020a). This approach shapes the SASE’s functionality, guiding its interactions in the DAC-HRC architecture to align with principles of both harm avoidance and proactive cooperation. This incorporation allows the Socially Adaptive Safety Engine to adapt its behavior dynamically, not only avoiding harm but also optimizing operation parameters such as speed, distance, and task allocation based on the unique context of each human worker. The Worker Model, integral to the contextual layer of the DAC-HRC architecture, helps personalize the interaction, treating each worker as a distinct individual with specific preferences and needs.

The Socially Adaptive Safety Engine module, in charge of providing a context-aware and personalized safety control system, spans across three layers of the DAC-HRC architecture. The SASE’s reactive layer integrates several homeostatic modules whose purpose is to monitor key aspects of the human-robot interaction. The goal of each homeostatic module is twofold: to keep its desired variable within the optimal range of operation, and to exert control when that variable trespasses the safe range. The current implementation comprises the homeostatic control of key proxemics variables in HRI, such as the human-robot interaction security distance, the robot movement speed, and the robot action execution speed. When any of those variables reach or trespass their threshold, the control response can be either a direct modification of the exceeded value -in the case of speed modulation-, or a command directed to stop the robot’s current action -in case the HRI distances are trespassed. For instance, if the actual detected distance between the human and the robot is below the desired safety value, the homeostatic control system will generate a stop signal and the robot will not move until the actual distance goes back to the desired range.

The Socially Adaptive Safety Engine’s adaptive layer is composed of the allostatic control module. This is the key mechanism by which the SASE can adapt the interaction of the robot to its changing environment. This module is in charge of the transformation of the environmental information provided by the contextual layer and modifying the desired parameters of the subsequent homeostatic regulatory mechanisms of the reactive layer. For instance, when the robot is handling a dangerous tool, the allostatic control module gets this information and adapts the desired safety HRI distance, as well as the speed at which the robot will operate when being close to a human.

The Socially Adaptive Safety Engine’s contextual layer deals with the integration of the relevant environmental, social, and material information that comes from other modules of the DAC-HRC architecture. It endows the SASE with context awareness. The constant integration of these different sources of information defines the specific context at every point in time, thus allowing the SASE to monitor and adapt the behavior of the robot to the changing conditions of its surroundings. For instance, the contextual layer can obtain information in real-time about the HRI preferences of the currently detected human worker, the risk level of the current robot action, and the information about the current tool being used by the robot (if any).

The incorporation of the reactive control systems inspired by the Pavlovian appetitive and aversive drives allows the Socially Adaptive Safety Engine to adapt its behavior dynamically, not only avoiding harm but also optimizing operation parameters such as speed, distance, and task allocation based on the unique context of each human worker. The worker model, integral to the contextual layer of the DAC-HRC architecture, helps personalize the interaction, treating each worker as a distinct individual with specific preferences and needs.

#### 2.2.4 Worker model

The Worker Model is composed of short-term and long-term memory buffers along with its reactive and adaptive input processing layers. The Worker Model’s reactive layer serves as a first data integration step, gathering information from several input sources, whereas its adaptive layer processes the raw data in order to produce new parameters that will be used by other modules of the Worker Model and the DAC-HRC architecture. The online information gathered by the Worker Model’s reactive layer is transiently stored in the short-term memory buffer before it is further processed by the adaptive Layer to generate new relevant information about the worker and their interaction with the system. For instance, the short-term memory can store the timings of past interactions during a disassembly step, while the adaptive layer generates estimates of current task duration based on this input. The type of input information gathered by the Worker Model can be divided into offline and online variables:

•Offline variables - This type of data is mostly static, as it will not vary throughout the session (e.g., age, gender, language, and interaction preferences). This information is acquired through preliminary questionnaires before engaging with the system and defines the profile of each user based on demographic information and her opinion towards robots.
• Online variables - Comprises all the relevant user data that is dynamically updated in real-time over the course of the interaction with the system. Integrates information about the position of the worker and their performance (e.g., current disassembly task, or estimated duration), as well as about the context in which the worker is embedded (e.g., current disassembly process, a Cell number, or location).

The technical implementation of the Worker Model is based on two main components: the Worker Model’s API and the Worker Model Database. The database implements the long-term memory component of the Worker Model. Its function is to store all the information related to each worker and to keep it up to date. It is deployed as a document-oriented database using MongoDB^1^, where each worker profile is stored as a unique document. Each worker model entry is initialized with the offline variables acquired from the worker profile and questionnaires. Additionally, it also stores the main statistics of each interaction between the worker and the DAC-HRC collaborative architecture that has been extracted by the Worker Model API, such as the expected task duration or the history of interactions with the tablet.

All the communications with the database are centrally controlled by the Worker Model’s API, which integrates the reactive and adaptive input processing layers along with the short-term memory component of the Worker Model. The API’s function is twofold: it performs the basic CRUD (create, read, update, and delete) operations that keep the database up to date, and it is in charge of filtering the online and state variables to produce the task- and interaction-relevant outputs of the Worker Model. The API is written in Python and communicates with the database using BSON as the data interchange format.

## 3 Results

In this section, we showcase the application of the DAC-HRC within the industrial context of the HR-Recycler hybrid recycling plant, highlighting the various functionalities of DAC-HRC that enhance human-robot collaboration in the recycling plant, specifically: (1) turn-taking human-robot collaborative interaction during the disassembly of a WEEE device, (2) error handling mechanisms personalized by past collaborative interactions, (3) adaptive and personalized safety measures for human-robot collaboration, and (4) gesture-based communication for goal-oriented collaboration. Each scenario was assessed during the disassembly of different WEEE devices, specifically: emergency lamps, computer towers, microwaves, and LCD displays. Importantly, trials to assess the robot’s autonomous disassembly capabilities were conducted prior to these tests; in all cases, the robot failed to successfully disassemble any device without human intervention or the application of the DAC-HRC. This failure serves as the reference process against which we benchmark our architecture’s performance. Furthermore, the experiments included various human participants to evaluate the architecture’s adaptability to different human actors and preferences. Given the nature of the experiments and the robot’s inability to complete tasks autonomously, we chose not to report these autonomous trials in the results section, focusing instead on the functionalities enabled by the DAC-HRC architecture.

### 3.1 Turn-taking human-robot collaborative interaction in the disassembly of a WEEE device

**FIGURE 4:**
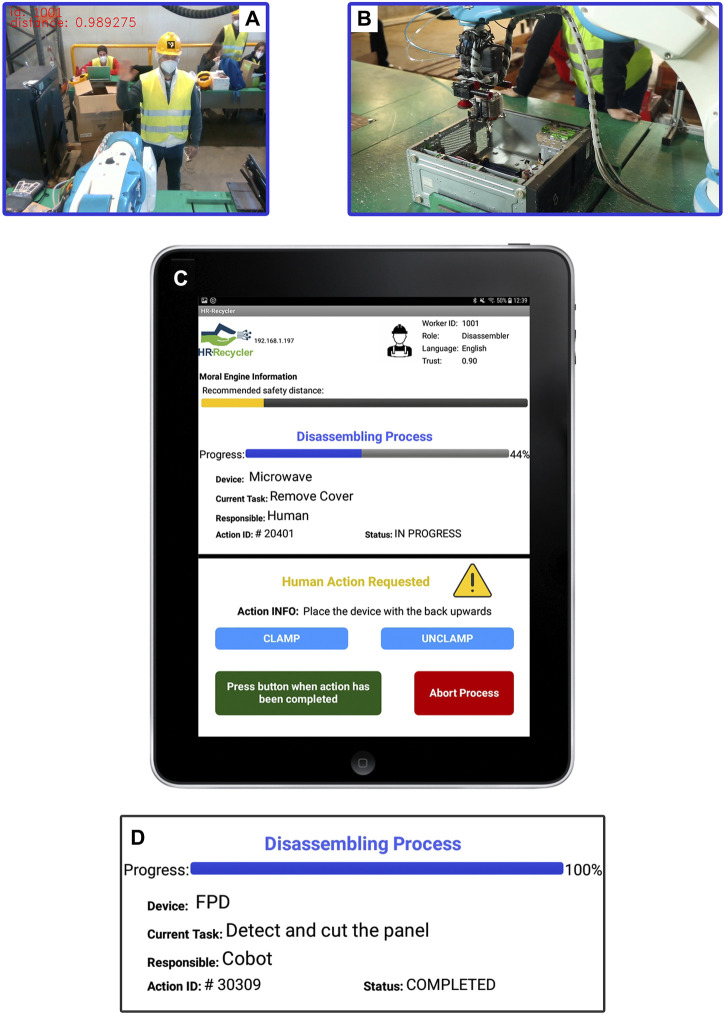
Human-Robot Collaborative Disassembly of a WEEE Device. **(A)** Vision module identifies a worker using their unique code. **(B)** DAC-HRC architecture adjusts to worker preferences, modulating robot behavior. **(C)** The IM app shows worker details and disassembly status and sends notifications if human input is required. **(D)** Task Planner updates after full disassembly of the WEEE device.

This use case describes the involvement of the DAC-HRC architecture during the collaborative disassembly of WEEE devices between a cobot and a human worker. Such a collaborative process begins when a human worker approaches the aCell. Once the worker enters the working area, they are recognized by the vision module that perceives their identity by decoding the unique fiducial code allocated in the workers’ helmets (Figure 4A). Then, using the identity of the worker, the Worker Model anonymously accesses their corresponding personal information and makes it available to the entire DAC-HRC architecture, so other cognitive modules can socially adapt to the current worker. This process reflects the principle of shared intentionality, as it involves a mutual understanding between the human and the cobot about the identity and role of the current worker. It also illustrates the principle of interdependence, as the overall disassembly performance depends on both the cobot and worker (Figure 4B).

The Interaction Manager receives and processes the worker’s personal information and provides them with immediate feedback about their detection by displaying such information through the IM app (Figure 4C). It is noteworthy that this information, and further notifications, are displayed meeting the worker language’s preferences. Importantly, the rapid communication between the vision modules and the Worker Model ensures that the social information considered by the cognitive architecture has real-time correspondence with the current human worker at the aCell.

In parallel, the cobot continues operating primarily guided by the goals imposed by the Task Planner. The succession of steps, as well as their status and the progress during the disassembling process, is also communicated to the human worker through the IM app (Figure 4C). However, as mentioned in the description of the Task Planner, the scheduling of disassembly steps is determined as a succession of states that ensures the task allocation (human or cobot) matches the worker’s skills and preferences. Thus, the optimal distribution of disassembly tasks leads to stable collaborative turn-taking dynamics, fostering predictability and facilitating the rapid acquisition of social conventions Hawkins and Goldstone (2016), Freire et al. (2020b).

Once the Task Planner has successfully overcome the robot’s assignments and reached an action that requires human intervention, this module interplays with the Interaction Manager to proactively interact with the human worker. As a result, the IM app sends a notification to the human worker describing the action to be performed (Figure 4C). Moreover, this notification enables the worker to control the clamping tool (see Figure 1) through the IM app when the device’s translation or reorientation is needed. Once the human intervention has been completed, a completion button must be pressed to allow the DAC-HRC architecture to carry on with the next step. Additionally, an abort option is available in cases where the human worker needs to stop the collaborative disassembly and finish on their own.

Finally, when both the robot and human’s disassembling actions have been completed, the Interaction Manager, in liaison with the Task Planner, informs the human worker about the completion of the disassembling process through the IM app (Figure 4D).

### 3.2 Error handling mechanisms personalized by past collaborative interactions

**FIGURE 5:**
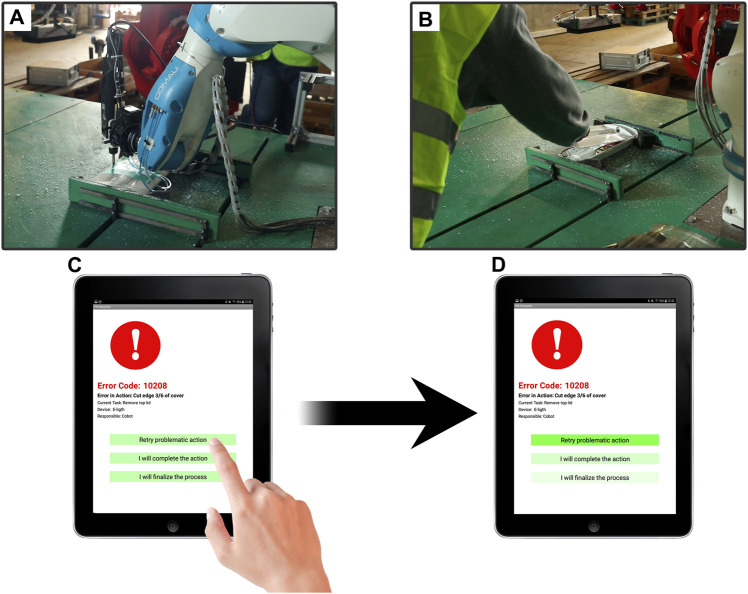
Personalized error-handling mechanisms during Human-Robot collaborative disassembly. **(A)** The complex coupling of both the aCel and the DAC-HRC architecture becomes a potential source of failure that needs to be addressed at the systems level. **(B)** Either when the cobot cannot complete a given disassembly action, or when is the worker’s turn to execute a step of the disassembly, the human worker can intervene safely. **(C)** IM app notification of an error during the disassembly providing the three different actions to overcome the error. **(D)** The IM app in liaison with the Interaction Learner provides an attentional bias towards the preferred error-handling options by modulating their visual saliency.

Beyond the complex interaction that DAC-HRC cognitive architecture maintains within its components, it is also in contact communication with other HR-Recycler sensory and control modules. This architecture’s complexity aims to both cope with the challenge of autonomously disassembling WEEE devices, but also ask for collaboration when unexpected issues prevent the optimal performance of the cobot’s assignments (Figure 5A).

To overcome these errors, the Task Planner, through the IM app, informs the human worker of any problematic action (i.e., any action that leads to errors) and provides three possible solutions. These options give the worker the possibility to (1) force the cobot to retry the problematic action, (2) inform the cobot that the worker will take care of the action (Figure 5B), or (3) to inform the cobot that the worker will take care of the remaining steps of the disassembly (Figure 5C).

Importantly, due to the involvement of the Interaction Learner module, this error-handling functionality becomes adaptive to the worker’s preferences by learning from previous interactions. Thus, if a worker exhibits consistent biases toward one of the options when handling Task Planner errors, the module memorizes these preferences and facilitates future decision-making by increasing the visual saliency of the previously preferred options (Figure 5D). This reflects the principles of shared intentionality and interdependence, as it involves a mutual understanding between the human and robot about handling errors and reliance on each other’s capabilities to resolve these errors and complete the disassembly process.

### 3.3 Adaptive and personalized safety measures for human-robot collaboration

**FIGURE 6:**
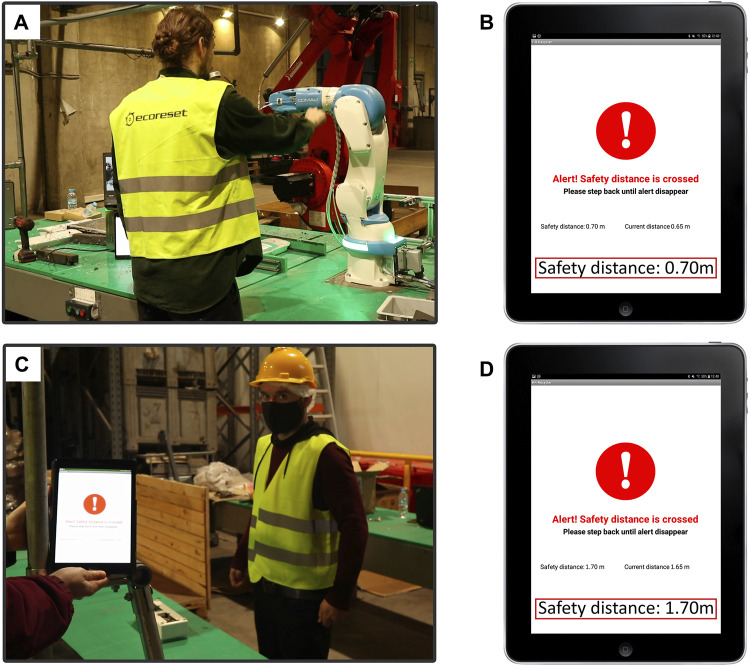
Adaptation of safety distance to human workers with different trust measures. **(A)** A worker with high trust in their robotic counterpart engages in the collaborative process of disassembling the WEEE device. Their high trust is considered by the Socially Adaptive Safety Engine which, accordingly, sets a short safety distance. Nonetheless, once this personalized safety distance is surpassed the robot comes back to its initial position and stops. **(B)** Surpass of the safety distance triggers an IM app alert notification. According to the worker’s high measure of trust, the personalized safety distance is set at 0.70 m **(C)** When a different worker reporting a lower measure of trust in their robotic counterpart enters the aCell, the Socially Adaptive Safety Engine recalculates the safety distance. As a result, the safety distance is enlarged and the human worker is not allowed to get so close to the cobot without triggering the safety alert. **(D)** IM app alert notification when the worker with lower trust surpasses the safety distance. Notice that it was enlarged to 1.70 m.

In parallel to the Human-Robot collaborative disassembling of WEEE devices, safety-related information is constantly monitored and processed to provide adaptive and personalized robot behavior. With this aim, once the computer vision module has detected and recognized a human worker at the aCell, the Socially Adaptive Safety Engine (SASE) draws its measure of trust from the Worker Model. In addition, the SASE updates the safety distance and robot’s speed according to the worker’s preferences (Figure 6). This process reflects the principles of shared intentionality and interdependence, as it involves a mutual understanding between the human and robot about the worker’s trust level and reliance on each other’s capabilities to maintain safety during the disassembly process.

Since the adaptation of the robot’s speed to the worker’s trust does not interfere with the worker’s performance, it has been designed to occur covertly and automatically. Thus, the robot’s speed is set at high levels when the current worker has reported high levels of trust in their robotic counterpart and decreases when a more distrusting worker enters the aCell.

However, aiming to ensure the integrity of the human workers, the normal turn-taking collaborative Human-Robot interaction can be interrupted when they surpass the safety distance (Figures 6A, B). This safety distance, as well as the robot’s speed, is initially personalized by the Socially Adaptive Safety Engine based on the trust information provided by the Worker Model. Thus, human workers with higher trust are allowed to get closer to the workbench while the cobot is carrying out its tasks. Nonetheless, when a more distrusting worker enters the aCell, this safety distance is extended ensuring both their physical integrity and physiological wellbeing (Figures 6C, D and Supplementary Videos S1 and S2). Importantly, even when workers report a maximum level of trust in robots, a minimum safety distance is set, following the international safety requirements for industrial robots ISO (2011, 2016). The real-time monitoring of the workers’ position relative to their personalized safety distance is accomplished by the DAC-HRC architecture due to the continuous communication between the SASE and the vision module, which provides the current worker’s location.

In cases where the worker has surpassed their personalized safety distance, the Socially Adaptive Safety Engine ensures their integrity by immediately stopping the cobot’s action. The trespassing of the safety distance is also reported to the Interaction Manager, which in turn notifies the human worker about their current location and the minimum distance they should keep to the cobot (Figures 6B, C). This alert remains displayed on the IM app until the worker gets back to respect their safety distance. Once the safety distance is reached again, the SASE’s alert disappears from the IM app and the cobot resumes its previous task.

### 3.4 Gesture-based communication for goal-oriented collaboration

Besides the direct input that human workers could provide to the DAC-HRC architecture through the IM app, vision modules recognize a set of gestures that enables multimodal communication and enhance human-robot interaction during collaborative disassembly.

**FIGURE 7:**
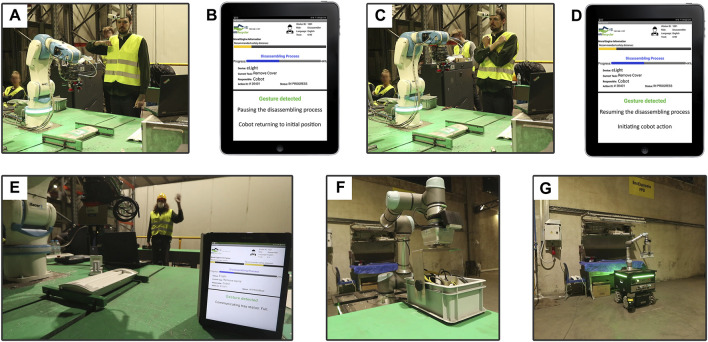
Human-Robot interaction based on gesture recognition. **(A)** Human worker performing the ‘stop’ gesture. **(B)** IM app notification for the recognition of the ‘stop’ gesture and showing information about the corresponding robot action: adopting its initial, default pose. **(C)** Human worker performing the ‘resuming’ gesture. **(D)** IM app notification for recognition of the ‘resuming’ gesture and showing information about the corresponding robot action, resuming the interrupted action. **(E)** Human worker performing the ‘wave’ gesture and IM app notifying about the recognition and meaning of the gesture: disposal tray is full. **(F)** Automated guided vehicle (AVG) robot picking up the full disposal tray from the aCell. **(G)** AVG robot leaving the full disposal tray in the removal area.

Unlike direct input through the IM app, which is dependent on specific events such as the requirement of human intervention or error-handling situations, gesture-based communication is available at any time during disassembly. That is, the workers can exert control over the collaborative process by performing predefined gestures that inform the DAC-HRC architecture to stop or resume the disassembling process, as well as informing that the disposal tray is full (Figure 7 and Supplementary Videos S3 and S4). Consequently, the worker also gets feedback about the detection of the recognized gesture through the IM app (Figures 7B, D, E). This reflects the principles of shared intentionality and interdependence, as it involves a mutual understanding between the human and robot about the meaning of different gestures, and a reliance on each other’s capabilities to interpret these gestures and respond appropriately (Figures 7F, G).

## 4 Discussion

This paper introduces the Distributed Adaptive Control-based Human-Robot Collaboration (DAC-HRC) architecture, a novel cognitive framework tailored for enhancing human-robot interactions within the dynamic and evolving landscape of Industry 4.0. Unlike traditional paradigms that promoted more static and rigid interactions, DAC-HRC represents a significant leap forward, integrating socially adaptive, flexible, and intuitive interaction schemes that cater specifically to the nuanced demands of industrial contexts. By leveraging novel Human-Robot Collaboration (HRC) strategies, such as gesture-based communication and user-context adaptation, DAC-HRC facilitates a more natural and efficient partnership between humans and non-humanoid robots, particularly within the challenging environment of electronic waste recycling.

At the heart of DAC-HRC are four main cognitive modules: the Task Planner, Socially Adaptive Safety Engine, Interaction Manager, and Worker Model. Each module is meticulously designed to operate across various timescales and abstraction levels, ensuring that the architecture can provide personalized adaptive collaboration that is sensitive to the unique needs of each human user. This modular design not only underscores the architecture’s flexibility but also its potential to enable seamless and organic human-robot interaction in complex and dynamic industrial scenarios.

Applied within the HR-Recycler environment, a hybrid recycling plant focused on the disassembly and recycling of Waste Electrical and Electronic Equipment (WEEE) devices, DAC-HRC’s capabilities were demonstrated through several pilot studies. These studies showcased the architecture’s ability to enhance human-robot collaboration through (1) adaptive turn-taking interactions, (2) personalized error-handling mechanisms, (3) dynamic safety measures, and (4) intuitive gesture-based communication. By addressing key collaboration aspects such as adaptation, safety, personalization, transparency, and real-time interaction, DAC-HRC proposes a new paradigm for human-robot collaboration in industrial settings.

In each of the outlined use cases, the DAC-HRC architecture demonstrates its capacity for real-time adaptive decision-making, informed directly by data gathered during human-robot interactions. For instance, in the collaborative disassembly of WEEE devices, the cobot’s operational speed and safety distances are dynamically adjusted based on the trust levels reported by the human workers. This socially adaptive mechanism ensures that interactions are tailored to individual comfort levels, thereby enhancing the safety and efficiency of the collaborative process. Similarly, the system’s ability to adapt to the language preferences of each worker, as identified through their unique fiducial codes, exemplifies how DAC-HRC leverages personal information to customize the interaction experience, ensuring clear and effective communication through the Interaction Manager. These adaptations, underpinned by principles of shared intentionality and interdependence, enable DAC-HRC to foster a cooperative environment that is responsive to the nuanced needs and preferences of human workers, significantly impacting the collaborative dynamics within the industrial setting of the HR-Recycler plant.

Despite the promising potential of DAC-HRC, current limitations such as the need for further validation and refinement, as well as the integration of additional cognitive modules for predictive task allocation and human behavior understanding, must be addressed.

The primary aim of this paper was to explore and demonstrate the feasibility and adaptability of the DAC-HRC cognitive architecture as a novel systems-level control paradigm for HRC, particularly within industrial settings. The focus of our pilot studies was to validate the cognitive architecture’s conceptual and functional capabilities, such as facilitating adaptive collaboration, enhancing safety measures, and implementing intuitive communication protocols.

Given the innovative and exploratory nature of this work, the emphasis was placed on qualitative assessments of the architecture’s integration and interaction dynamics within the HR-Recycler environment, rather than on quantitative performance metrics. This approach aligns with the initial stages of deploying such complex systems, where understanding the system’s behavior, adaptability, and potential for enhancing human-robot collaboration takes precedence. Therefore, while the inclusion of performance metrics is undoubtedly valuable for evaluating HRC systems, the current phase of this research was focused on establishing a foundational understanding and proof of concept of the DAC-HRC architecture. Future work should focus on incorporating quantitative performance metrics to rigorously evaluate the architecture’s effectiveness and efficiency in enhancing human-robot collaboration.

Recognizing the importance of these human-centric factors, future research should also incorporate more formal evaluations of the human aspects of collaboration. This includes assessing the user experience, perceived usefulness, and mental load using standardized tools like the NASA-TLX, alongside additional metrics that can provide a more comprehensive understanding of the human-robot interaction dynamics. These future studies aim to balance the focus between technical innovation and human factors, ensuring that advancements in HRC systems like DAC-HRC not only meet technical and safety requirements but also align with human workers’ needs and preferences for a truly collaborative and supportive work environment.

The interdisciplinary nature of DAC-HRC’s development, drawing from cognitive science, robotics, and human-robot interaction research, is a testament to its innovative approach to solving complex HRC challenges. This cross-disciplinary collaboration has enabled the creation of an architecture that not only meets the technical requirements of industrial applications but also aligns with the cognitive and social dynamics of human-robot interaction.

The collaborative entity of DAC-HRC termed the aCell, symbolizes a distributed cognitive organism akin to an ant colony, where cognitive processes are shared among agents to achieve collective goals. This analogy is rooted in the notions of extended cognition Clark and Chalmers (1998) and liquid brains Solé et al. (2019), which describe how cognitive processes can be distributed across multiple agents in a system, rather than being confined to a single individual. It highlights the importance of designing distributed hybrid collaborative systems that leverage the complementary strengths of humans and robots. By fostering shared control and distributed agency, DAC-HRC paves the way for innovative approaches to human-robot collaboration that can significantly impact Industry 4.0 and beyond.

In an ant colony, for example, no single ant possesses the entire knowledge of the colony’s activities. Instead, each ant contributes to the collective intelligence of the colony through its individual actions and interactions with other ants. Similarly, in an aCell, the human and cobot work together as a cohesive unit, with each contributing their unique skills and capabilities to the collective performance of the task at hand.

This perspective offers valuable insights for designing distributed hybrid collaborative systems. For instance, it suggests that we should focus not only on the individual capabilities of humans and robots but also on how they can best interact and coordinate their actions to achieve shared goals. This could involve developing natural language understanding methods that enable humans and robots to share information more effectively Dong et al. (2019), Thomason et al. (2019), or designing control algorithms that allow robots to adapt their behavior based on the expected actions and intentions of their human partners Shum et al. (2019), Lake et al. (2017), Freire et al. (2023).

Moreover, by integrating principles of shared intentionality and interdependence, the DAC-HRC architecture provides a robust foundation for future endeavors in human-robot collaboration across industrial settings and beyond, aiming to enhance the cognitive and communicative dynamics of collaborative tasks. This principled framework encourages the creation of more socially-aware, adaptable hybrid systems capable of supporting nuanced human-robot interactions in diverse environments. For example, in manufacturing, such insights could guide the development of cobots engineered to proactively respond to human workers’ needs, facilitating real-time adjustments to workflow tasks or machine pacing to alleviate worker fatigue or optimize productivity. Similarly, in healthcare, DAC-HRC’s approach could lead to assistive robots that offer tailored support to patients or healthcare providers, learning from each interaction to improve responsiveness and adapt behavior based on individual preferences or emotional cues. Looking ahead, DAC-HRC’s expansion into other sectors such as logistics and warehouse management promises to leverage these insights further, driving the creation of more efficient, empathetic, and adaptable collaborative systems that elevate the efficacy of human-robot partnerships in any context. By capitalizing on the complementary strengths of humans and robots in this way, we can create hybrid collaborative systems that enable them to work together more effectively and efficiently.

In sum, DAC-HRC’s commitment to enhancing the collaborative bond between humans and robots through adaptation, safety, personalization, and transparency sets a new blueprint for future hybrid industrial collaborative efforts. The architecture’s modular and flexible framework aims to advance the efficiency and efficacy of human-robot partnerships, providing valuable insights for both industrial applications and the broader human-robot interaction research community. As we continue to explore and expand the capabilities of DAC-HRC, it stands as a testament to the potential of cognitive architectures to revolutionize the way humans and robots work together, paving the way for more responsive, understanding, and cooperative collaborative systems.

## Data Availability

The original contributions presented in the study are included in the article/Supplementary Material, further inquiries can be directed to the corresponding authors.

## Footnotes

1
https://www.mongodb.com
