# Supplementary figures and images for: Socially adaptive cognitive architecture for human-robot collaboration in industrial settings
Source: Front Robot AI. 2024 Jun 10;11:1248646. doi: 10.3389/frobt.2024.1248646 (PMC11194424; doi:10.3389/frobt.2024.1248646)

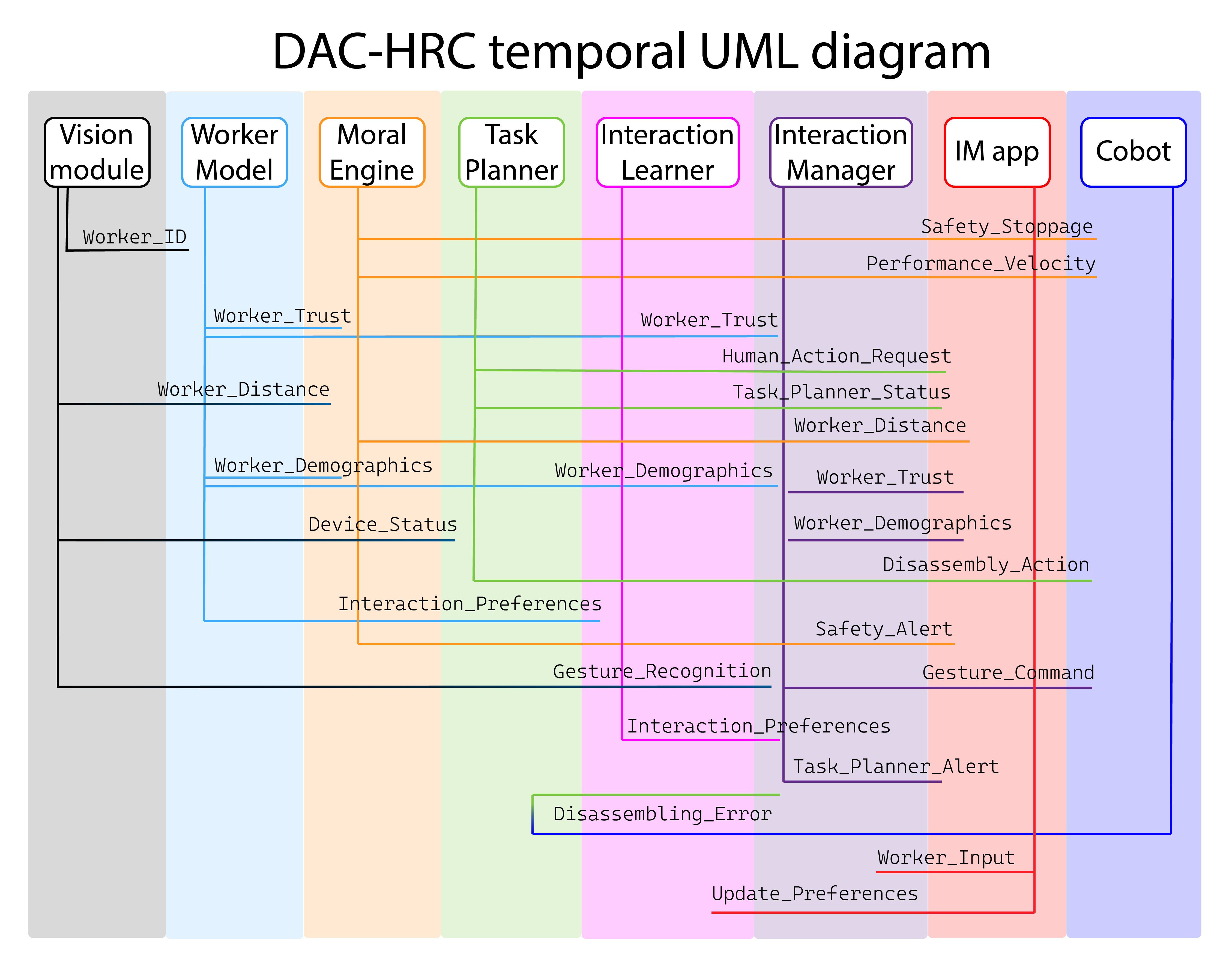

Supplement: Supplementary file 4 [file Image1.PNG]
